# Supplementary material for: Zero-temperature glass transition in two dimensions
Source: Nat Commun. 2019 Apr 3;10:1508. doi: 10.1038/s41467-019-09512-3 (PMC6447585; doi:10.1038/s41467-019-09512-3)
Supplement: Supplementary file 1 — Supplementary Information [file 41467_2019_9512_MOESM1_ESM.pdf]

Supplementary Information for "Zero-temperature glass transition in two dimensions"

Berthier et al.

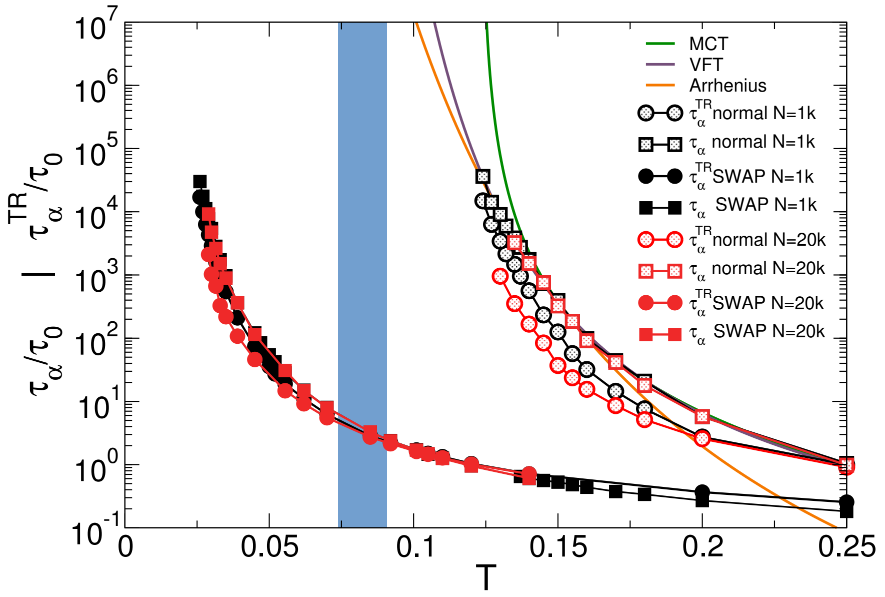

Supplementary Figure 1 : Relaxation times as a function of the temperature for both normal and SWAP dynamics. The y-axis is rescaled by the relaxation time of normal dynamics at the onset temperature $\tau_\alpha(T_{\text{onset}} = 0.25) = \tau_0 = 2592$ MCsteps. Empty (full) symbols indicate normal (SWAP) dynamics. Circles (squares) denote results for translational (orientational) relaxation times τ_α^{TR} (τ_α) for $N = 1000$ and $N = 20000$ systems. The MCT, VFT and Arrhenius fits (see text) are given as green, purple and orange solid lines, respectively. These fits help estimate the glass ceiling region, *i.e.* the lower bound for the region accessible in equilibrium experiments, which is denoted as a blue box.

Supplementary Note 1. STRUCTURAL CORRELATIONS

In the main text, we show that ξ_{PTS} increases as temperature decreases. Ref. [1], however, showed that for some computational models made of polydisperse particles, correlation lengths related to the degree of order present increase faster than ξ_{PTS}. In particular, Ref. [1] analyzed the two-points positional and bond-orientational correlations, paying particular attention to the radial decay of the functions $g(r) - 1$ and $g_6(r)/g(r)$, respectively

Results for these two quantities are reported in Supplementary Figure 2. Here, following Ref. [1], Delaunay neighbors are obtained from a radical Voronoi tessellation. Both functions exhibit clear peaks at distances corresponding to the correlation shells, but their temperature evolution is relatively mild. We fit the peak points with an exponential function of the form $C_{s,6}\exp(-r/\xi_{s,6})$ in order to extract a correlation function both for positional ξ_s and bond-

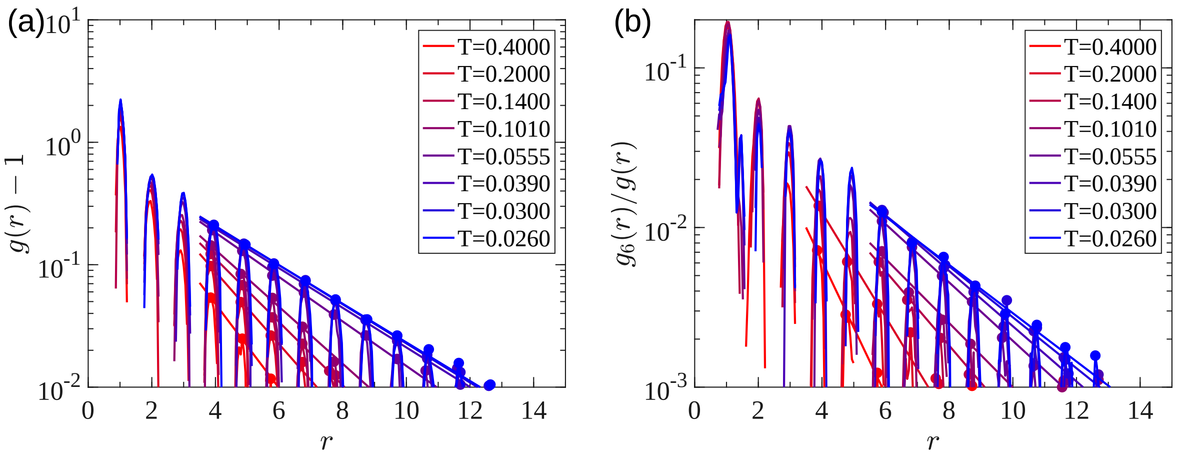

Supplementary Figure 2 : Two-point (a) positional and (b) bond-orientational correlation functions. Colors denote different temperatures from red (high) to blue (low). Peak maxima are fitted with an exponential form, $C_{\rm s,6}\exp(-r/\xi_{\rm s,6})$, in order to extract positional and bond-orientational static correlation lengths $\xi_{\rm s}$ and ξ_6, respectively.

orientational ξ_6 correlations. The temperature evolution of the resulting static correlation lengths is shown in Supplementary Figure 3 together with that of $\xi_{\rm PTS}$. Over the whole temperature range, we observe an increase by a factor ≈ 2.2 and ≈ 2.7 for $\xi_{\rm s}$ and ξ_6 with saturation at low temperature, which is considerably smaller than the factor of ≈ 5.1 increase observed for $\xi_{\rm PTS}$. Coupled with the additional verifications for potential crystallization and fractionation, this result rules out the presence of significant structural order in our system, even at extremely low temperatures. Our observations are also remarkably different from those of Ref. [1]; they show that good glass formers are not affected by increases in positional and bond-orientational order.

Supplementary Note 2. CONFIGURATIONAL ENTROPY

The configurational entropy, $s_{\rm conf}$, is defined as

$$s_{\rm conf} = s_{\rm tot} - s_{\rm glass}, \tag{1}$$

where $s_{\rm tot}$ and $s_{\rm glass}$ are the total entropy and the entropy of a typical glass state, respectively. We separately measure $s_{\rm tot}$ and $s_{\rm glass}$ by thermodynamic integration based on the scheme developed in Ref. [2].

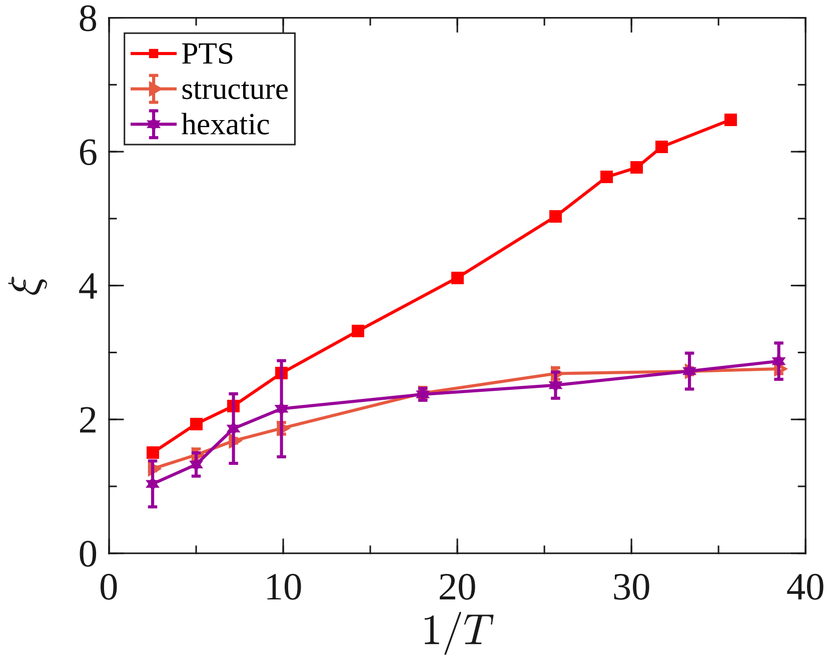

Supplementary Figure 3 : Growth of different static correlation lengths with temperature: positional order length, $\xi_{\rm s}$, bond-orientational order length, ξ_6, and point-to-set length, $\xi_{\rm PTS}$ (see Supplementary Note 3). Errorbars for $\xi_{\rm s}$ and ξ_6 correspond to the 95% confidence interval of the associated fitting. The increase of the first two lengths is mild compared to that of $\xi_{\rm PTS}$.

A. Setting

Consider a M-component polydisperse system. (A system with $M = N$ is said to have a continuous polydispersity.) If N_m is the number of particles of the m-th species, then the fraction of the m-th species is $X_m = N_m/N$, and hence $\sum_{m=1}^{M} N_m = N$ and $\sum_{m=1}^{M} X_m = 1$. For simplicity, we set all particles masses to unity. We denote particle positions as $\mathbf{r}^N = (\mathbf{r}_1, \mathbf{r}_2, \cdots, \mathbf{r}_N)$, and the set of their diameter as $\Sigma^N = \{\sigma_1, \sigma_2, \cdots, \sigma_N\}$. In order to consider permutations of particle diameters as additional degrees of freedom, we introduce a permutation π to the set Σ^N. A specific sequence of particle diameters is denoted Σ_π^N, e.g., $\Sigma_\pi^N = (\sigma_3, \sigma_8, \sigma_5, \cdots)$. A total of $N!$ possible such permutations exists, and for a system with continuous polydispersity, all such permutations are distinguishable.

The system potential energy, U, depends both on particle positions $\mathbf{r}^N$ and on the permutation π, and is thus formally denoted $U(\Sigma_\pi^N, \mathbf{r}^N)$. For notational simplicity, we write $U(\mathbf{r}^N) = U(\Sigma_{\pi^*}^N, \mathbf{r}^N)$ for the reference system with $\Sigma_{\pi^*}^N$. The resulting canonical partition

function at inverse temperature $\beta = 1/T$ is

$$\mathcal{Z} = \frac{1}{N!}\sum_{\pi}\frac{1}{\Pi_{m=1}^{M} N_m!\Lambda^{Nd}}\int_V \mathrm{d}\mathbf{r}^N e^{-\beta U(\Sigma_\pi^N, \mathbf{r}^N)}, \tag{2}$$

where $\Lambda = \sqrt{2\pi\beta\hbar^2}$ is the thermal de Broglie wavelength with the unit mass. Without loss of generality, we set the Planck constant $\hbar = 1$. Note that Eq. (2) should be distinguished from the conventional partition function, Z, in which only particle positions $\mathbf{r}^N$ are degrees of freedom,

$$Z = \frac{1}{\Pi_{m=1}^{M} N_m!\Lambda^{Nd}}\int_V \mathrm{d}\mathbf{r}^N e^{-\beta U(\mathbf{r}^N)}. \tag{3}$$

The following subsections describe how Eq. (2) can be used to compute both the total and the glass entropies.

B. Total entropy

The partition function $\mathcal{Z}$ in Eq. (2) for the target system $\beta U(\Sigma_\pi^N, \mathbf{r}^N)$ reduces to the conventional partition function Z without permutations in Eq. (3), because diameter permutations are always compensated by position permutations in absence of constraint, i.e.,

$$\mathcal{Z} = \frac{1}{N!}\sum_{\pi}\frac{1}{\Pi_{m=1}^{M} N_m!\Lambda^{Nd}}\int_V \mathrm{d}\mathbf{r}^N e^{-\beta U(\Sigma_\pi^N, \mathbf{r}^N)} = \frac{1}{\Pi_{m=1}^{M} N_m!\Lambda^{Nd}}\int_V \mathrm{d}\mathbf{r}^N e^{-\beta U(\mathbf{r}^N)} = Z\,. \tag{4}$$

The total entropy computation is therefore equivalent to what has been observed in previous studies [3, 4].

Using a high-temperature $\beta \to 0$ ideal gas as an exactly solvable reference system, we perform a thermodynamic integration over (inverse) temperature up to the target temperature β,

$$s_{\mathrm{tot}} = \frac{(d+2)}{2} - \ln\rho - \ln\Lambda^d + \beta e_{\mathrm{pot}}(\beta) - \int_0^\beta d\beta' e_{\mathrm{pot}}(\beta') + s_{\mathrm{mix}}^{(M)}, \tag{5}$$

where $s_{\mathrm{mix}}^{(M)} = \frac{1}{N}\ln\left(N!/\Pi_{m=1}^{M} N_m!\right)$ is the ideal gas mixing entropy per particle and $e_{\mathrm{pot}}(\beta)$ is the average potential energy per particle. The integration in Eq. (5) requires special care, because $e_{\mathrm{pot}}(\beta)$ diverges in the high-temperature limit [3, 4]. We sidestep the difficulty by introducing an intermediate temperature β_0 that separates the very high temperature regime, $\beta' \in [0, \beta_0]$, from the rest, $\beta' \in (\beta_0, \beta]$. We thus write

$$I \equiv \int_0^\beta d\beta' e_{\mathrm{pot}}(\beta') = \int_0^{\beta_0} d\beta' e_{\mathrm{pot}}(\beta') + \int_{\beta_0}^{\beta} d\beta' e_{\mathrm{pot}}(\beta') \equiv I_{\mathrm{F}} + I_{\mathrm{N}}, \tag{6}$$

where $I_{\rm N}$ is obtained by usual thermodynamic integration, and $I_{\rm F}$ is obtained by fitting the $e_{\rm pot}(\beta)$ to a polynomial, and then analytically integrating the resulting function [3, 4]. The specific polynomial form we use for the high-temperature expansion of a system of soft spheres with interaction potential $v(r) \propto r^{-n}$ (in d dimensions) is

$$e_{\rm pot}(\beta) = A\beta^{(d/n)-1} + B\beta^{(2d/n)-1} + C\beta^{(3d/n)-1} + D\beta^{(4d/n)-1} + \cdots, \tag{7}$$

where the constants A, B, C, and D are determined by fitting, as in Supplementary Figure 4(a). Using Eqs. (6) and (7), we then get

$$I_{\rm F} = \int_0^{\beta_0} d\beta' e_{\rm pot}(\beta') = \frac{n}{d}A\beta_0^{d/n} + \frac{n}{2d}B\beta_0^{2d/n} + \frac{n}{3d}C\beta_0^{3d/n} + \frac{n}{4d}D\beta_0^{4d/n} + \cdots. \tag{8}$$

which only depends on the fit parameters, A, B, C, and D. Supplementary Figure 4(b) presents the results for $s_{\rm tot} - s_{\rm mix}^{(M)}$ obtained by this procedure. Comparing results for systems with $N = 1000$ and $N = 20000$ confirms the absence of size dependence.

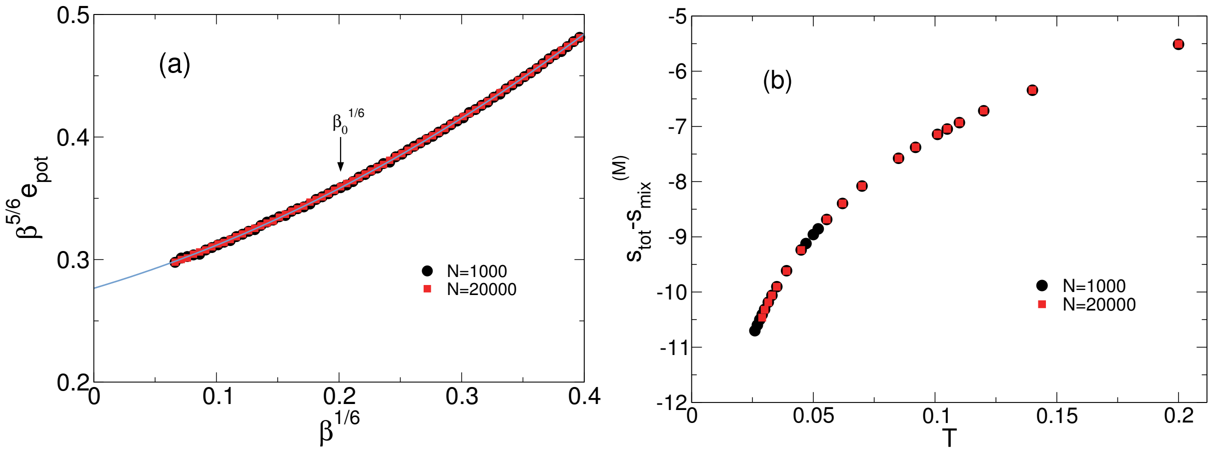

Supplementary Figure 4 : (a) High-temperature average potential energy results, $\beta^{5/6}e_{\rm pot}(\beta)$ for N=1000 (black circles) and N=20000 (red squares) systems, along with the resulting fitting form (blue line) with parameters A, B, and C as in Eq. (8). Including D in the fit has no noticeable numerical impact. The vertical arrow denotes our choice of $\beta_0^{1/6} = 0.2013$. The total entropy results are unchanged for any reasonable choice of β_0. (b) The resulting temperature dependence of $s_{\rm tot} - s_{\rm mix}^{(M)}$.

C. Glass entropy

We evaluate the entropy of glass states by Frenkel-Ladd (FL) thermodynamic integration [5–8], which requires imposing a harmonic potential with spring constant α on particle positions. The process then entails integrating the long-time limit of the mean-squared displacement starting from a strong $\alpha_{\max}$, at which the system behaves as an Einstein solid, and reaching a weak $\alpha_{\min}$, at which the system is self caged. More specifically, we set

$$\beta U_\alpha(\Sigma_\pi^N, \mathbf{r}^N, \mathbf{r}_0^N) = \beta U(\Sigma_\pi^N, \mathbf{r}^N) + \alpha \sum_{i=1}^{N} |\mathbf{r}_i - \mathbf{r}_{0i}|^2, \tag{9}$$

where $\mathbf{r}_0^N$ is the template configuration from the equilibrium configuration of the target system.

As for the total entropy, we start from the partition function in Eq. (2) for the glass state,

$$\mathcal{Z}_\alpha = \frac{1}{N!} \sum_\pi \frac{N!}{\Pi_{m=1}^M N_m! \Lambda^{Nd}} \int_V \mathrm{d}\mathbf{r}^N e^{-\beta U_\alpha(\Sigma_\pi^N, \mathbf{r}^N, \mathbf{r}_0^N)}. \tag{10}$$

Note that the numerator of Eq. (10) is now multiplied by $N!$, because a given template configuration, $\mathbf{r}_0^N$, selects a single glass basin from the position phase space, while there exists $N!$ identical such choices, generated by permuting $\mathbf{r}_0^N$. Note also that the presence of the template configuration $\mathbf{r}_0^N$ prevents diameter permutations from being compensated by position permutation. The identity in Eq. (4) therefore does not hold in the glass state. The integration limit, $\lim_{\alpha_{\min}\to 0}$, also requires special conceptual and practical considerations. Although for FL integration of a crystal $\alpha_{\min}$ is chosen to be infinitesimally small, here an additional constraint is that the system should remain within a glass basin and should thus not melt. The practical implementation of this constraint is detailed below.

We compute the entropy $s_\alpha = \beta e_{\mathrm{tot},\alpha} - \beta f_\alpha$, where $e_{\mathrm{tot},\alpha}$ is the total energy and $f_\alpha = -(\beta N)^{-1} \ln \mathcal{Z}_\alpha$ is the free energy. The glass entropy of the target system is then

$$s_{\mathrm{glass}} = \lim_{\alpha_{\min}\to 0} \overline{s_{\alpha_{\min}}}, \tag{11}$$

where $\overline{\cdots}$ here denotes averaging over template configurations $\mathbf{r}_0^N$.

For convenience, we also define the following statistical averages,

$$\langle(\cdots)\rangle_{\alpha}^{\mathrm{T,S}} = \frac{\frac{1}{N!}\sum_{\pi}\int_V \mathrm{d}\mathbf{r}^N(\cdots)e^{-\beta U_\alpha(\Sigma_\pi^N,\mathbf{r}^N,\mathbf{r}_0^N)}}{\frac{1}{N!}\sum_{\pi}\int_V \mathrm{d}\mathbf{r}^N e^{-\beta U_\alpha(\Sigma_\pi^N,\mathbf{r}^N,\mathbf{r}_0^N)}}, \tag{12}$$

$$\langle(\cdots)\rangle_{\alpha}^{\mathrm{T}} = \frac{\int_V \mathrm{d}\mathbf{r}^N(\cdots)e^{-\beta U_\alpha(\mathbf{r}^N,\mathbf{r}_0^N)}}{\int_V \mathrm{d}\mathbf{r}^N e^{-\beta U_\alpha(\mathbf{r}^N,\mathbf{r}_0^N)}}, \tag{13}$$

$$\langle(\cdots)\rangle_{\beta}^{\mathrm{S}} = \frac{\frac{1}{N!}\sum_{\pi}(\cdots)e^{-\beta U(\Sigma_\pi^N,\mathbf{r}_0^N)}}{\frac{1}{N!}\sum_{\pi} e^{-\beta U(\Sigma_\pi^N,\mathbf{r}_0^N)}}, \tag{14}$$

where the superscripts denote statistical averages over positions (T) and permutations (S), evaluated by Monte Carlo (MC) simulations with standard translations and diameter swaps, respectively. Note that any diameter permutation can be expressed as the product of the swaps of two diameters, hence permutation-phase space is properly sampled by swap MC simulations.

Following the conventional Frenkel-Ladd prescription [5] for Eq. (10), we obtain

$$s_{\mathrm{glass}} = \frac{d}{2} - \ln\Lambda^d - \frac{d}{2}\ln\left(\frac{\alpha_{\max}}{\pi}\right) + \lim_{\alpha_{\min}\to 0}\int_{\alpha_{\min}}^{\alpha_{\max}} \mathrm{d}\alpha\Delta_{\alpha}^{\mathrm{T,S}} + s_{\mathrm{mix}}^{(M)} - \overline{\mathbf{s}_{\mathrm{mix}}(\mathbf{r}_0^N,\beta)}, \tag{15}$$

where $\Delta_{\alpha}^{\mathrm{T,S}}$ are constrained mean-squared displacements

$$\Delta_{\alpha}^{\mathrm{T,S}} = \frac{1}{N}\overline{\left\langle \sum_{i=1}^{N}|\mathbf{r}_i - \mathbf{r}_{0i}|^2 \right\rangle_{\alpha}^{\mathrm{T,S}}}, \tag{16}$$

and $\mathbf{s}_{\mathrm{mix}}(\mathbf{r}_0^N,\beta)$ is a mixing entropy contribution defined by

$$\mathbf{s}_{\mathrm{mix}}(\mathbf{r}_0^N,\beta) = -\frac{1}{N}\ln\left(\frac{1}{N!}\sum_{\pi} e^{-\beta\left[U(\Sigma_\pi^N,\mathbf{r}_0^N)-U(\mathbf{r}_0^N)\right]}\right). \tag{17}$$

This generalization of the standard FL integration method to systems with continuous polydispersity includes two novel physical features. First, the mean-squared displacement $\Delta_{\alpha}^{\mathrm{T,S}}$ has to be evaluated by MC simulations of both translational and swap displacements, and is thus generally distinct from the standard mean-squared displacement, $\Delta_{\alpha}^{\mathrm{T}}$. Because $\Delta_{\alpha}^{\mathrm{T,S}}$ accounts for the non-vibrational contributions due to diameter permutations as well as for the purely vibrational contribution, $\Delta_{\alpha}^{\mathrm{T,S}} \geq \Delta_{\alpha}^{\mathrm{T}}$. Including the non-vibrational contribution also markedly improves the estimation of the glass entropy [2, 9], as we will see below. Second, the expression contains terms related to the mixing entropy, $s_{\mathrm{mix}}^{(M)} - \overline{\mathbf{s}_{\mathrm{mix}}}$. The diverging term, $s_{\mathrm{mix}}^{(M=N)} = \ln N - 1 \to \infty$, in Eq. (15) then exactly cancels the corresponding term in s_{tot} in Eq. (5). The remaining mixing entropy contribution, $\overline{\mathbf{s}_{\mathrm{mix}}}$ in s_{conf}, is finite even for systems

with continuous polydispersity. Therefore, with this scheme continuous polydispersity does not present any conceptual or technical difficulty [2].

The key remaining tasks in order to compute $s_{\rm glass}$ involve measuring the mixing entropy contribution $\overline{\mathrm{s}_{\rm mix}}$ and integrating $\Delta_\alpha^{\rm T,S}$. Both are detailed below.

Mixing entropy $\overline{\mathrm{s}_{\rm mix}}$

The mixing entropy contribution, $\overline{\mathrm{s}_{\rm mix}}$, is determined by thermodynamic integration,

$$\overline{\mathrm{s}_{\rm mix}(\mathbf{r}_0^N,\beta)} = \frac{1}{N}\int_0^\beta \mathrm{d}\beta' \overline{\Delta U_{\rm mix}(\mathbf{r}_0^N,\beta')}, \tag{18}$$

where $\Delta U_{\rm mix}$ is a potential energy difference defined by

$$\Delta U_{\rm mix}(\mathbf{r}_0^N,\beta') = \left\langle U(\Sigma_\pi^N,\mathbf{r}_0^N)\right\rangle_{\beta'}^{\rm S} - U(\mathbf{r}_0^N). \tag{19}$$

In practice, to get $\Delta U_{\rm mix}(\mathbf{r}_0^N,\beta')$ the system is gradually heated from the target temperature β to an infinite temperature $\beta \to 0$ using MC simulations with a fraction $p_{\rm swap}=1$ of the diameter swaps. Particles are thus kept at the same position as in the template configuration $\mathbf{r}_0^N$. As shown in Supplementary Figure 5(a), $\overline{\Delta U_{\rm mix}}/N$ takes very small values at large β, but sharply increases upon approaching $\beta \to 0$. Note that $\overline{\Delta U_{\rm mix}}/N$ remains finite at $\beta \to 0$, hence so does $\overline{\mathrm{s}_{\rm mix}}$. The resulting $\overline{\mathrm{s}_{\rm mix}}$ then increases slightly as temperature decreases, as seen in Supplementary Figure 5(b). We confirm the absence of size dependence by comparing results for systems with $N=1000$ and $N=20000$.

Integration of $\Delta_\alpha^{\rm T,S}$

Starting from $\alpha=\alpha_{\rm max}$, we perform MC simulations with decreasing α in steps of $\delta(\log_{10}\alpha) \simeq 0.18-0.4$. For each data point, we perform $\tau = 2\times10^4 - 2\times10^6$ MC steps, measuring $\Delta_\alpha^{\rm T,S}$ only in the second half of the simulation. Supplementary Figure 6(a) shows the evolution of $\Delta_\alpha^{\rm T,S}$ with α. At large α, the system is an Einstein solid with $\Delta_\alpha^{\rm T,S}=1/\alpha$, but upon decreasing α, $\Delta_\alpha^{\rm T,S}$ plateaus. The system is then self caged. Further decreasing α, however, makes the harmonic constraint too weak to prevent the glass state from melting, thus implicitly defining $\alpha_{\rm min}$. The ensuing particle diffusion explains the upturn of $\Delta_\alpha^{\rm T,S}$. In order to perform the integration in Eq. (15), a practical manipulation of the limit must be

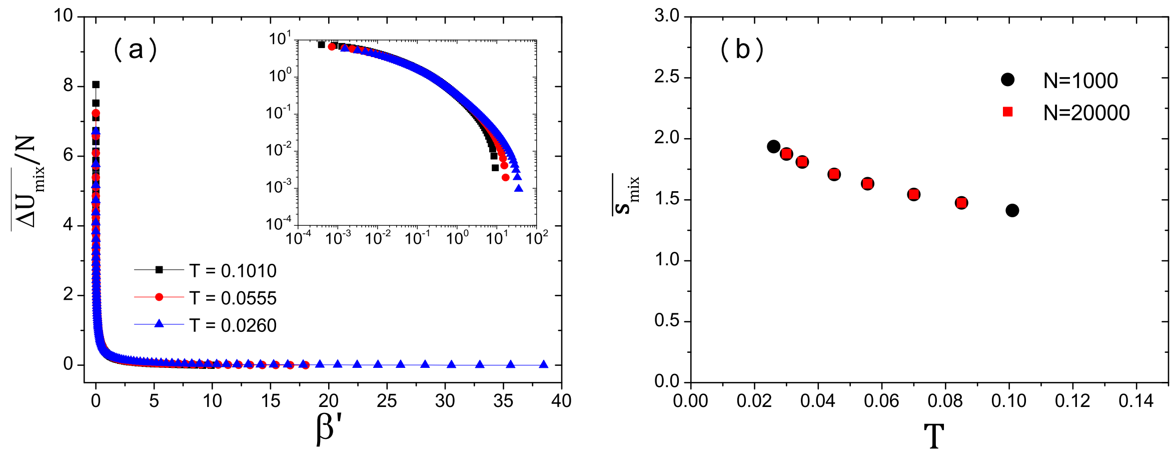

Supplementary Figure 5 : (a) Temperature evolution of $\overline{\Delta U_{\rm mix}(\mathbf{r}_0^N, \beta')}$ for several equilibrium template configurations for $N = 1000$. (Inset) Same data on a logarithmic scale. (b) $\overline{\mathbf{s}_{\rm mix}}$ obtained by Eq. (18) as a function of the temperature for N=1000 (black circles) and N=20000 (red squares) systems.

used for $\alpha < \alpha_{\rm min}$. We consider

$$\lim_{\alpha_{\rm min}\to 0} \int_{\alpha_{\rm min}}^{\alpha_{\rm max}} {\rm d}\alpha \Delta_\alpha^{\rm T,S} \simeq \alpha_{\rm min}\Delta_{\alpha_{\rm min}}^{\rm T,S} + \int_{\alpha_{\rm min}}^{\alpha_{\rm max}} {\rm d}\alpha \Delta_\alpha^{\rm T,S}$$

$$= \alpha_{\rm min}\Delta_{\alpha_{\rm min}}^{\rm T,S} + (\ln 10) \int_{\log_{10}\alpha_{\rm min}}^{\log_{10}\alpha_{\rm max}} {\rm d}(\log_{10}\alpha)\alpha\Delta_\alpha^{\rm T,S}. \quad (20)$$

While $\alpha_{\rm max}$ should straightforwardly be chosen in the Einstein solid regime, e.g., we use $\alpha_{\rm max} \simeq 1 \times 10^7$, the choice of $\alpha_{\rm min}$ is not unambiguous. Based on the above discussion, we understand that $\alpha_{\rm min}$ should be within the plateau regime of $\Delta_\alpha^{\rm T,S}$, where $\Delta_\alpha^{\rm T,S}$ does not depend on τ. In order to identify the regime of proper equilibration in the plateau region, the τ-dependence of $\Delta_\alpha^{\rm T,S}$ is presented in Supplementary Figure 7 (a, b). As seen in these figures, if α is too small, $\Delta_\alpha^{\rm T,S}$ increases at large τ. The shaded region denotes the regime in which the time needed to obtain well averaged observables has no detectable τ dependence. This corresponds to the regime within which $\alpha_{\rm min}$ can be safely chosen. The choice of $\alpha_{\rm min}$ nonetheless affects $s_{\rm glass}$, especially at high temperatures, where a plateau never fully forms. The systematic uncertainty associated with this choice is captured by the errorbars for $s_{\rm glass}$ in the shaded region, $\alpha_{\rm min} \in [10.1, 40.5]$, of Supplementary Figure 6(a). The edges of the errorbar in Supplementary Figure 6(c) correspond to $s_{\rm glass}$ extracted from the two extremes of the shaded region, $\alpha_{\rm min} = 10.1$ and 40.5. As expected, these error bars become smaller as temperature decreases, thus validating our choice of $\alpha_{\rm min}$. Since $s_{\rm conf}$

in the main text depends on the chosen $\alpha_{\min}$ in the determination of s_{glass}, we display the errorbars corresponding to s_{conf} from $\alpha_{\min}$-values chosen inside the plateau region, in the same way as in Supplementary Figure 6(c). In the main text, s_{conf} using the above scheme is called FL.

Effetc of Mermin-Wagner (MW) fluctuations

Note that because $\Delta_\alpha^{\mathrm{T,S}}$ essentially coincides with the plateau height of the dynamically measured mean-squared displacement, one may also expect MW fluctuations to contribute significantly [10]. To assess the relevance of MW fluctuations, we consider the system size dependence of $\Delta_\alpha^{\mathrm{T,S}}$ in Supplementary Figure 6(b). $\Delta_\alpha^{\mathrm{T,S}}$ systematically increases with increasing N ($N = 300 - 20000$) at very small α, as expected from the presence of MW fluctuations [10]. However, there is no finite size effect in the range of our interest, $\alpha_{\min}$, suggesting that imposing a weak harmonic constraint suppresses MW fluctuations without disturbing the overall thermodynamics of the system. This process is thus akin to the effect of pinning a few percent of the particles as was reported in Ref. [11].

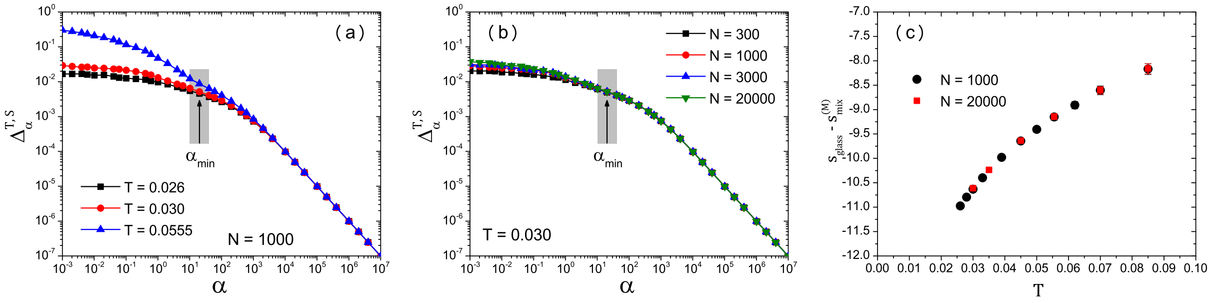

Supplementary Figure 6 : (a) Mean-squared displacement $\Delta_\alpha^{\mathrm{T,S}}$ in the Frenkel-Ladd construction at several temperatures for $N = 1000$ with $\tau = 2 \times 10^4$. The shaded region denotes the potential range for $\alpha_{\min}$, and the arrow the specific choice of $\alpha_{\min} = 20.3$. (b) System size dependence of $\Delta_\alpha^{\mathrm{T,S}}$ for $T = 0.030$ with $\tau = 2 \times 10^4$. (c) s_{glass} obtained by Eq. (15) using $\alpha_{\min} = 20.3$ for $N = 1000$ and 20000. The diverging mixing entropy term, $s_{\mathrm{mix}}^{(M)}$, is subtracted. The span of the errorbars corresponds to s_{glass} for $\alpha_{\min} \in [10.1, 40.5]$.

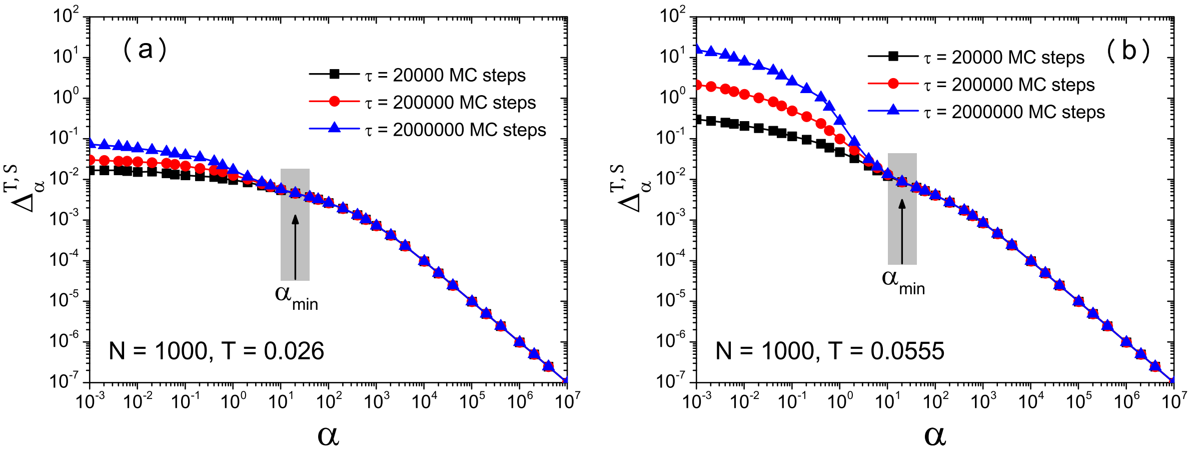

Supplementary Figure 7 : Timescale dependence of $\Delta_\alpha^{\mathrm{T,S}}$ for $N = 1000$ at (a) $T = 0.026$ and (b) $T = 0.0555$. For both temperatures, no τ dependence is observed up to $\alpha_{\mathrm{min}} \in [10.1, 40.5]$ (shaded region).

D. Potential energy landscape approach

We also consider an alternate approach for estimating s_{conf} based on the potential energy landscape (PEL) [12]. In this approach, the glass entropy is obtained from information about the inherent structures (IS) of the glass state. In order to evaluate the impact of polydispersity on s_{conf}, we employ an effective M^*-component approximation as in Ref. [13]. This approach provides an effective mixing entropy $s^*_{\mathrm{mix}} = s^{(M^*)}_{\mathrm{mix}}$. (The numerical determination of M^* is explained below.) We then compute the glass entropy s_{glass} by $s_{\mathrm{glass}} = s_{\mathrm{harm}} + s_{\mathrm{anh}}$, where s_{harm} and s_{anh} are the harmonic vibrational entropy and its anharmonic correction, respectively [12]. The harmonic term is computed as

$$s_{\mathrm{harm}} = \frac{1}{N} \left\langle \sum_{a=1}^{d(N-1)} \{1 - \ln(\beta\hbar\omega_a)\} \right\rangle_{\mathrm{IS}}, \tag{21}$$

where $\langle \cdots \rangle_{\mathrm{IS}}$ is an average over IS configurations obtained by the conjugate gradient method and $\omega_a = \sqrt{\lambda_a/m}$ is the square root of eigenvalue λ_a of the Hessian of this IS. Supplementary Figure 8(a) shows s_{harm} as a function of T for $d = 2$.

The anharmonic contribution to the potential energy is $e_{\mathrm{anh}}(T) = e_{\mathrm{pot}}(T) - e_{\mathrm{IS}}(T) - \frac{d}{2}T$, where e_{IS} is the inherent structure energy, and the last term is the harmonic contribution to the energy. From $e_{\mathrm{anh}}(T)$, we also have

$$s_{\mathrm{anh}}(T) = \int_0^T dT' \frac{1}{T'} \frac{\partial e_{\mathrm{anh}}(T')}{\partial T'}, \tag{22}$$

where we used the fact that the system is perfectly harmonic at low T, i.e., $s_{\rm anh}(T=0)=0$. A low-temperature expansion, $e_{\rm anh}(T) = \sum_{k=2} c_k T^k$, has T-independent coefficients, c_k. Substituting this expansion into Eq. (22) gives

$$s_{\rm anh}(T) = \sum_{k=2} \frac{k}{k-1} c_k T^{k-1}. \tag{23}$$

The fit of $e_{\rm anh}$ with parameters c_2 and c_3 is shown in Supplementary Figure 8(b), and the resulting $s_{\rm harm} + s_{\rm anh}$ is shown in Supplementary Figure 8(a). The resulting anharmonic contribution is $|s_{\rm anh}| < 0.1$ in the temperature range of interest.

MW fluctuations effects

The glass entropy measured using the PEL approach also is not affected by MW fluctuations. Consider first the mean-squared displacement of standard solids, $\langle |\mathbf{u}|^2 \rangle$. For a monodisperse crystalline solid, one can write

$$\langle |\mathbf{u}|^2 \rangle = \frac{dk_{\rm B}T}{m} \int_{2\pi c/L}^{\infty} {\rm d}\omega \frac{g(\omega)}{\omega^2}, \tag{24}$$

where $g(\omega)$ and c are the vibrational density of states and the velocity of sound, respectively. Because one expects a Debye scaling $g(\omega) \propto \omega^{d-1}$ at low ω, in $d=2$, $\langle |\mathbf{u}|^2 \rangle \sim \ln L \to \infty$ diverges in the thermodynamic limit. Writing Eq. (21) using the density of state formalism,

$$s_{\rm harm} = d \int_{2\pi c/L}^{\infty} {\rm d}\omega g(\omega) \left\{ 1 - \ln(\beta \hbar \omega) \right\} , \tag{25}$$

by contrast, in $d=2$ gives the L-dependent term, $\frac{\ln L}{L^2}$, that vanishes as the system size increases. Additionally, $s_{\rm anh}$ does not depend on system size because e and $e_{\rm IS}$ (and thus $e_{\rm anh}$) display no system-size dependence at large enough L. The glass entropy, $s_{\rm harm} + s_{\rm anh}$, is therefore system-size independent and hence unaffected by MW fluctuations.

Determination of M^*

The effective component, M^*, is determined based on the potential energy landscape. As explained in Ref. [13], M^* should be such that (i) particle diameter swaps within a single effective species leave the potential energy basin unaffected, and (ii) particle diameter swaps between different species drive the system out of the original basin. To determine M^* in practice, we prepare equilibrium configurations of the original continuously polydisperse system characterized by the distribution $f(\sigma)$. We then decompose $f(\sigma)$ into M species

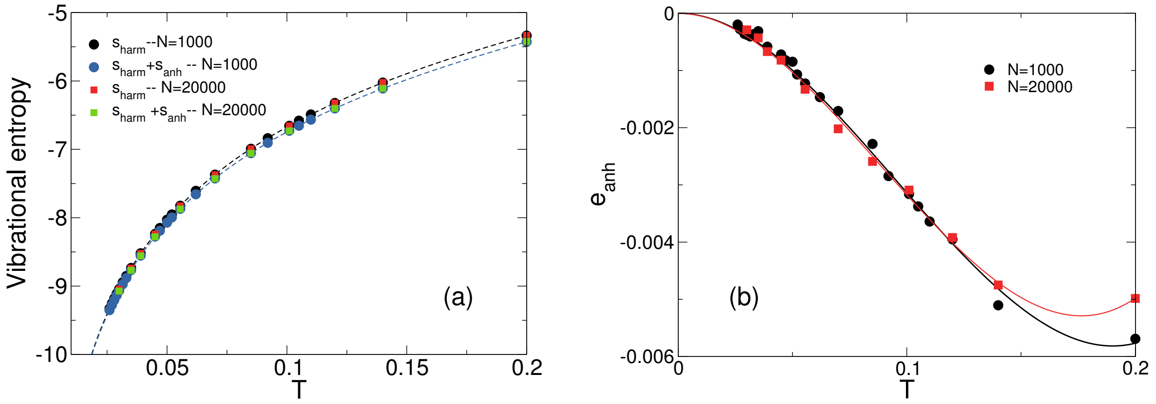

Supplementary Figure 8 : (a) Vibrational entropy results for $d = 2$ including the harmonic contribution $s_{\rm harm}$ and also its anharmonic correction $s_{\rm anh}$. (b) The anharmonic contribution of the potential energy $e_{\rm anh}$.

(from $M = 1$ to 100), dividing $f(\sigma)$ into equal intervals $\Delta\sigma = (\sigma_{\rm max} - \sigma_{\rm min})/M$, such that each species occupies more or less the same fraction of the total volume. For a given M value, we systematically perform diameter swaps within each species. We repeat such diameter swap N times so that most particles experience the swap. We then quench the obtained configuration to its IS, monitoring whether the system lands in a different basin (for $M < M^*$) or not (for $M > M^*$) by measuring $e_{\rm IS}$ as a function of M (or $x = \log_{10} M$) [see Supplementary Figure 9(a)].

At large $x = \log_{10} M$, we observe nearly constant values of $e_{\rm IS}^{(M)} \simeq e_{\rm IS}^{(M=N)}$, which means that the swap of the diameters within each M species marginally affects the system. After the diameter swaps, the system thus essentially remains in the original basin. However, with decreasing M, $e_{\rm IS}^{(M)}$ starts to increase significantly from $e_{\rm IS}^{(M=N)}$. This observation indicates that at smaller M, the impact of particle swaps is so strong that the original basin is destroyed, and the system moves to another basin.

From the $e_{\rm IS}$ vs. $x = \log_{10} M$ plot, the clear crossover between large and small M behaviors determines M^* as the intersection of two linear fits as shown in Supplementary Figure 9(a). We show the resulting $s^*_{\rm mix} = s^{(M^*)}_{\rm mix}$ as a function of the temperature in Supplementary Figure 9(b). We confirm the absence of size dependence by comparing results for systems with $N = 1000$ and $N = 20000$.

We also employ an exponential fit as an alternative way to extract M^* from the crossover.

We use the following exponentially decaying function: $e_{\rm IS}(x) = e_{\rm IS}^{(M=N)} + A\exp[-(x-x_0)/B]$, where $x_0 = \log_{10} M_0$ is the starting point of the exponential fitting, and A and B are fitting parameters. We set $M_0 = 4$ thus $x_0 = 0.602$. The exponential functional form precisely captures the data points as shown in Supplementary Figure 9(a). Here we define $x^* = \log_{10} M^*$ by the location where the exponential function decays sufficiently, i.e., $(e_{\rm IS}(x^*) - e_{\rm IS}^{(M=N)})/A = C$, where C is an arbitrary small value. We set $C \simeq 0.2$ so that M^* by this exponential scheme corresponds to the one by the intersection of the two linear fits described above for $T = 0.12$ where the linear fit scheme is good. As shown in Supplementary Figure 9(b), the resulting $s^*_{\rm mix} = s^{(M^*)}_{\rm mix}$ by the exponential fit eventually follows similar temperature dependence of the linear fit, suggesting robustness of our numerical determination of M^*. In the main text, $s_{\rm conf}$ using $s^*_{\rm mix}$ from the linear and exponential fit schemes are called PEL1 and PEL2, respectively.

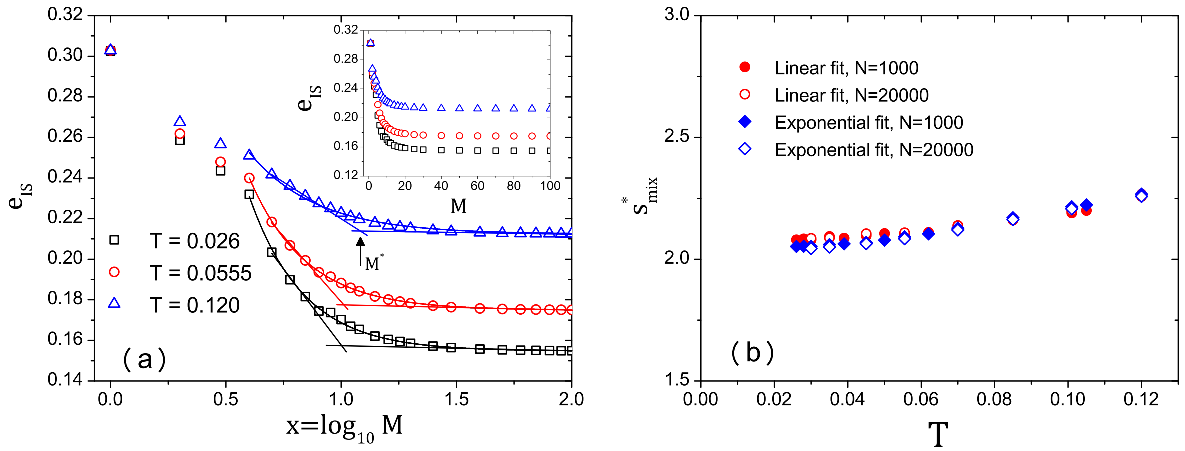

Supplementary Figure 9 : (a) $e_{\rm IS}$ vs. $x = \log_{10} M$ plot for the determination of M^* for $N = 1000$. The horizontal axis is the logarithmic plot. The vertical arrow represents the M^* value determined by the intersection of the two straight lines. The exponential fit is also shown. Inset: The horizontal axis is linear. (b) $s^*_{\rm mix} = s^{(M^*)}_{\rm mix}$ as a function of T for M^* determined by the linear fit and exponential fit for $N = 1000$ and 20000.

E. Absence of finite size effect in configurational entropy

We compile the entropy data in Figs. 4(b), 5(b), 6(c), 8(a), and 9(b) into a configurational entropy plot in Supplementary Figure 10. In order to assess presence of the finite size effect,

we present $s_{\rm conf}$ for $N = 1000$ and 20000. There is no noticeable finite size effect down to $T = 0.030$. The linear box length L for $N = 1000$ is around 30, which is still quite larger than the maximum point-to-set length in our simulation ($\xi_{\rm PTS} \simeq 7$). This observation is consistent with the absence of the finite size effect. Thus we conclude that $N = 1000$ data provide reliable estimations toward $T \to 0$ as shown in the main text.

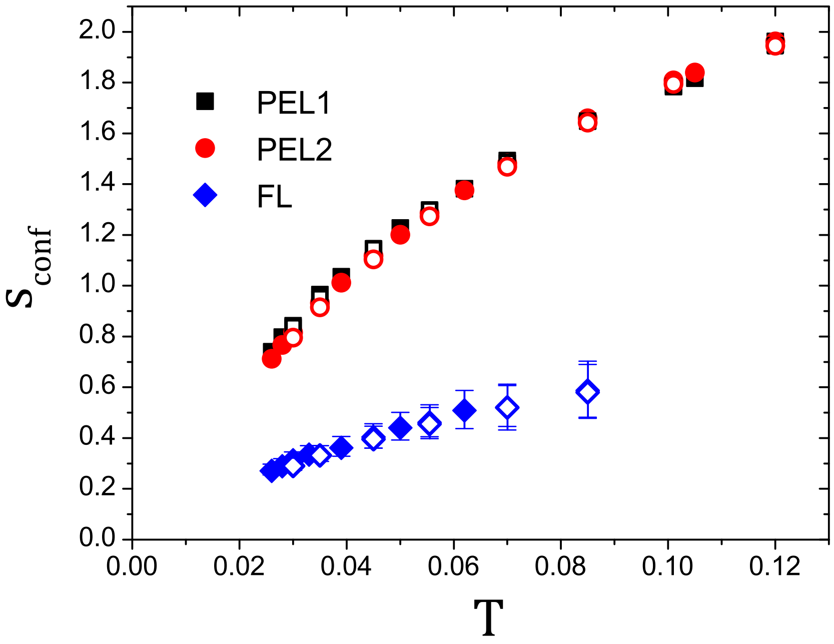

Supplementary Figure 10 : The configurational entropy $s_{\rm conf}$ obtained by the potential energy landscape approach (PEL1 and PEL2) and the Frenkel-Ladd thermodynamic integration approach (FL) for $N = 1000$ (filled points) and 20000 (empty points). The errorbars for FL correspond to $s_{\rm conf}$ from $\alpha_{\rm min}$-values chosen inside the plateau region of $\Delta_\alpha^{\rm T,S}$, in the same as as in Supplementary Figure 6(c).

Supplementary Note 3. POINT-TO-SET (PTS) CORRELATIONS

This section reports the setups and the results for point-to-set observables in soft disks; results for hard disks are reported in subsection Supplementary Note 3 E.

A. PTS observables

Similarity between two configurations within the cavity is characterized by the cavity core overlap, q_{c}, computed as in Refs. [14–17]. (i) We assign a local overlap value to each particle through the overlap estimator function $w(z) \equiv \exp\left[-\left(\frac{z}{b}\right)^2\right]$ with $b = 0.2$; (ii) we perform a linear interpolation through a Delaunay tessellation to define a continuous overlap field; and (iii) we measure the cavity core overlap by taking the average of the field values within the radius $r_{\mathrm{c}} = 1.0$ from the cavity center, evaluated by MC integration with 10^3 points.

For each temperature T and cavity radius R, the PTS correlation function

$$Q_{\mathrm{PTS}}(R;T) = [\langle q_{\mathrm{c}}\rangle]|_{T,R}\,, \tag{26}$$

is evaluated by disorder-averaging–denoted $[\ldots]$–over 100 cavity centers (200 for $0.0315 \leq T \leq 0.039$ and 300 for $T = 0.028$) and, within each cavity, thermal-averaging–denoted $\langle\ldots\rangle$–over s_{prod} pairs of equilibrated configurations (see subsection Supplementary Note 3 B).

One way to extract the PTS correlation length is through the compressed exponential fit,

$$Q^{\mathrm{fit}}_{\mathrm{PTS}}(R;T) = A\exp[-\left\{R/\xi^{\mathrm{fit}}_{\mathrm{PTS}}(T)\right\}^{\gamma}] + Q^{\mathrm{bulk}}_{\mathrm{PTS}}(T)\,, \tag{27}$$

with the bulk value, $Q^{\mathrm{bulk}}_{\mathrm{PTS}}$, evaluated by taking 10^5 pairs of independent configurations in bulk samples. Note that differently from Ref. [14–17], the compression exponent γ [see Supplementary Figure 11(a)] is here not fixed but treated as an additional fit parameter. Its value ranges roughly from 2 to 5 from high to low temperatures. Another definition of the PTS length, $\xi^{\mathrm{th}}_{\mathrm{PTS}}$, is given by the relation $Q^{\mathrm{fit}}_{\mathrm{PTS}}(\xi^{\mathrm{th}}_{\mathrm{PTS}};T) - Q^{\mathrm{bulk}}_{\mathrm{PTS}} \equiv e^{-1}$. A third estimate comes from the peak location of the PTS susceptibility [14] [see Supplementary Figure 11(b)],

$$\chi_{\mathrm{PTS}}(R;T) = [\langle q_{\mathrm{c}}^2\rangle - \langle q_{\mathrm{c}}\rangle^2]|_{T,R}\,. \tag{28}$$

Specifically the peak location, $\xi^{\mathrm{peak}}_{\mathrm{PTS}}$, is estimated through polynomial extrapolation of five maximal values. All three estimates qualitatively support the conclusion that the PTS correlation length diverges upon approaching $T = 0$ in $d = 2$ [see Supplementary Figure 11(c) and subsection Supplementary Note 3 E for hard disks]. In the main text, the threshold estimate $\xi^{\mathrm{th}}_{\mathrm{PTS}}$ is employed.

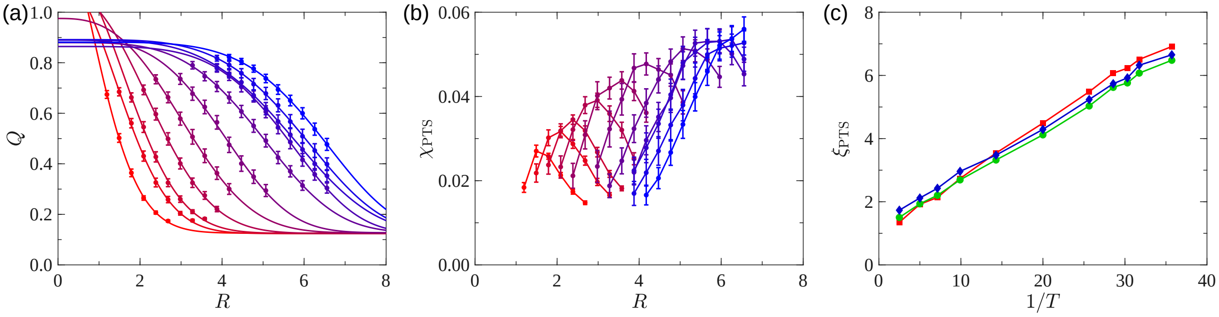

Supplementary Figure 11 : (a) Radial decay of the cavity PTS correlation at $T = 0.400$, 0.200, 0.140, 0.101, 0.070, 0.050, 0.039, 0.035, 0.033, 0.0315, and 0.028 (from red to blue) for soft disks. The errorbars correspond to the 95% confidence intervals estimated over cavity-to-cavity variations. Solid lines are fits to a compressed exponential. (b) PTS susceptibilities with cavity radius R. The errorbars are the same as in (a). Solid lines are guides for the eyes. (c) PTS lengths $\xi_{\mathrm{PTS}}^{\mathrm{fit}}$ (red-square), $\xi_{\mathrm{PTS}}^{\mathrm{th}}$ (green-circle), and $\xi_{\mathrm{PTS}}^{\mathrm{peak}}$ (blue-diamond) as a function of the inverse temperature for soft disks. The clear linear growth suggests that $T_{\mathrm{K}} = 0$ and that the RFOT exponent $\theta = 1 = \frac{d}{2} = d - 1$ in $d = 2$.

B. PTS equilibration

In order to properly and efficiently sample the cavity configurations, we employ a parallel-tempering scheme [18, 19] adapted to the cavity sampling as in Refs. [14] with varying temperatures and shrinking factors (T_a, λ_a) for replicas $a = 1, \ldots, n$, where $a = 1$ corresponds to the original ensemble. Within each replica, for a cavity containing N_{cav} mobile particles, one MC sweep entails N_{cav} MC trial moves consisting of 80% local displacements–with its length uniformly sampled from $l \in [0, 0.15]$– and 20% particles identity swaps. For cavity sizes $R > 2.0$, in order to accelerate runs, swap moves are attempted only for particle pairs with diameter difference $\lambda_a|\sigma_i - \sigma_j| < 0.20$. A replica-identity swap is then attempted every 1000 MC sweeps on average.

As in Ref. [14], we impose the linear relation between replica temperatures and shrinking factors as $\frac{T_a - T_1}{T_{\mathrm{dec}} - T_1} = \frac{\lambda_a - \lambda_1}{\lambda_{\mathrm{dec}} - \lambda_1}$ with T_{dec} and λ_{dec} chosen appropriately (see Supplementary Tables 1-11). The chosen shrinking factors, $\{\lambda_a\}_{a \geq 2}$, ensure sufficient replica-swap rates. In order to achieve this sampling, replicas are added one by one, with $\lambda_1 = 1 > \lambda_2 > \ldots > \lambda_n$, each time targeting a replica-swap acceptance rate of $\sim 20\%$ [17]. This process is stopped upon reaching $\lambda_n < \lambda_{\mathrm{dec}}$. In Supplementary Tables 1-11, the average number of replicas

used, $n_{\mathrm{ave}} = [n]$, is recorded for each given temperature and radius.

The quality of the equilibration within each cavity is assessed from monitoring the convergence of two preparation schemes [14, 20]: one starting from the original configuration and the other starting from a randomized configuration prepared by running 10^4 MC sweeps with shrunk and heated cavity particles, with $(\lambda, T) = (0.6, 0.5)$. Convergence is deemed achieved when

$$\langle q_{\mathrm{c}}^{\mathrm{on}} \rangle \equiv \frac{1}{s_{\mathrm{prod}}} \sum_{s=s_{\mathrm{eq}}+1}^{s_{\mathrm{eq}}+s_{\mathrm{prod}}} q_{\mathrm{c}}^{\mathrm{on}}\left(t_{\mathrm{rec}} s\right) \tag{29}$$

obtained through both approaches lie within ± 0.1 of each other for each cavity. Here $q_{\mathrm{c}}^{\mathrm{on}}(t)$ is the cavity core overlap between the original configuration and the equilibrated configuration after t MC sweeps, recorded each $t_{\mathrm{rec}} = 10^4$ MC sweeps. The first s_{eq} configurations are discarded, and thermal averages are taken over the following s_{prod} configurations. With our choice of parallel-tempering parameters (see Supplementary Tables 1-11), for all temperatures and radii, at least 96% of all cavities pass the convergence test. Averaging over cavities results in an even closer agreement between the two schemes, i.e., overlap estimates converge to within ± 0.01.

In obtaining PTS correlation functions and PTS susceptibility in Eqs. (26) and (28), respectively, we evaluate core cavity overlaps for s_{prod} pairs of configurations obtained through the two different schemes.

C. Glassiness

As detailed in Ref. [16], PTS observables and equilibration diagnose glassiness by accessing information about the underlying free-energy landscape. On the static side, the probability distribution function of cavity core overlaps exhibits broad fluctuations at the PTS length scale, with bimodal distribution in the deeply glassy regime (see Supplementary Figure 12). This nontrivial signature of confinement in turn leads to a peak in the PTS susceptibility and to a nonconvex dependence of the PTS correlation, as functions of the cavity radius R (see Supplementary Figure 11) [14]. In nonglassy systems, by contrast, these nontrivial behaviors are absent [16].

Proper sampling within cavity confinement grows increasingly challenging as R decreases. Without parallel-tempering, the relaxation time explodes for decreasing R (see Supplemen-

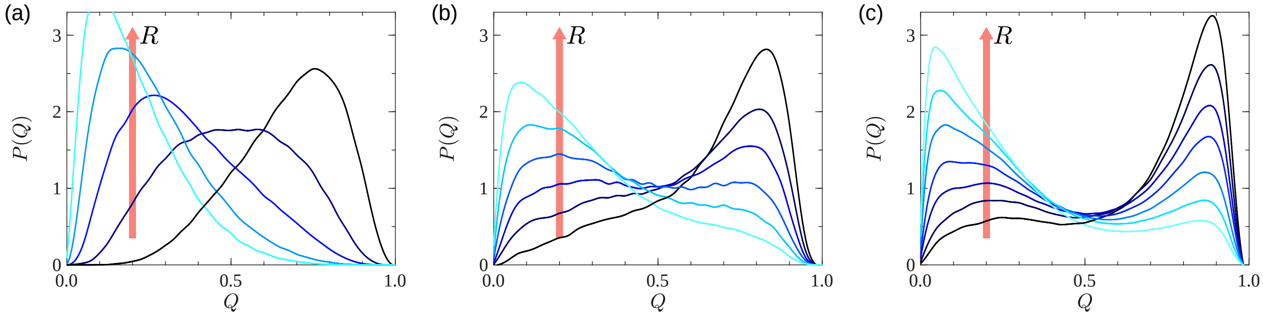

Supplementary Figure 12 : Disorder-averaged probability distribution function of core overlap $P(q_c)$, at $T = 0.400$ for radii $R = 1.2, 1.5, \ldots, 2.4$ (a), $T = 0.070$ for $R = 2.7, 3.0, \ldots, 4.2$ (b), and $T = 0.035$ for $R = 4.8, 5.1, \ldots, 6.6$ (c). As temperature decreases, the bimodal structure becomes more pronounced.

tary Figure 13). This dynamical observation also bears out that the slowdown in our polydisperse soft-disk system is triggered by the rugged free-energy landscape characteristic of glassiness.

D. Finite-size effect

Throughout the paper and this section, we have presented the results for PTS observables with cavities curved out of the bulk systems with $N = 1000$ particles. In Supplementary Figure 14, results for configurations with $N = 300$ and $N = 8000$ are presented for $T = 0.101$ and 0.050. No significant finite-size dependence of the results is observed.

R	1.2	1.5	1.8	2.1	2.4	2.7
n_{ave}	5.16	5.67	5.72	5.18	4.44	4.02
λ_{dec}	0.700	0.750	0.800	0.850	0.900	0.920
T_{dec}	0.400	0.400	0.400	0.400	0.400	0.400
s_{eq}	1000	1000	1000	1000	1000	1000
s_{prod}	4000	4000	4000	4000	4000	4000

Supplementary Table 1 Cavity PTS measurement parameters $T = 0.400$, with 100 cavities.

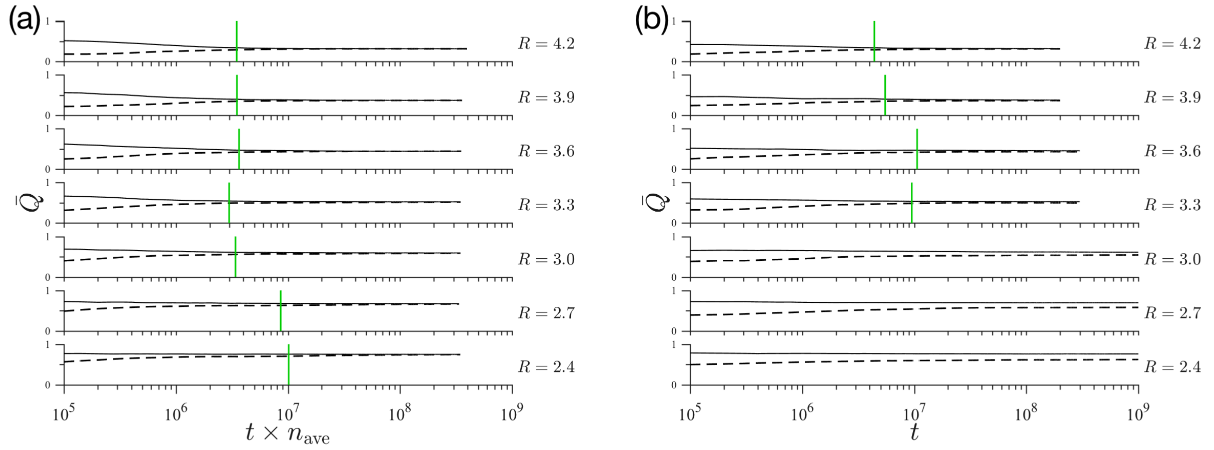

Supplementary Figure 13 : Running average of core overlaps [14], $\bar{Q}(t) \equiv \frac{1}{(t/t_{\rm rec})} \sum_{s=1}^{(t/t_{\rm rec})} q_{\rm c}^{\rm on}(t_{\rm rec}s)$, after t MC sweeps from both the original (solid lines) and a randomized (dashed lines) configurations at $T = 0.070$ for a cavity of radius R, averaged over 100 such cavities. Each green vertical line denotes an estimate of an equilibration times, here defined to be the time beyond which the difference between the running averages $\bar{Q}$ from two schemes converges within 0.05. (a) With parallel tempering. In order to compare appropriate computational times, x-axis is multiplied by the average number of replicas, $n_{\rm ave}$ (see Table 5). (b) Without parallel tempering. The equilibration time rapidly grows as the cavity radius shrinks, which is interpreted as a finite-size echo of a glass transition [14]. For instance, for $R \leq 3.0$ equilibration is not attained even after 10^9 MC steps.

E. Results for hard disks

For the point-to-set length measurement, we also study a two-dimensional hard-disk model, for which the pair interaction is zero for non-overlapping particles and infinite otherwise. The system has the same size distribution $f(\sigma)$ and size polydispersity δ as the soft-disks described in the main text. Given these parameters, the system is then uniquely characterized by its area fraction $\varphi = \pi N \overline{\sigma^2}/(4V)$, and we frequently report the data using the reduced pressure $Z = P/(\rho k_{\rm B} T)$, where ρ, $k_{\rm B}$, and T are the number density, Boltzmann constant and temperature, respectively. Without loss of generality, we set k_B and T to unity for the hard-disks. The pressure P is calculated from the contact value of the pair correlation function properly scaled for a polydisperse system [21]. We use $N = 1000$ for this model.

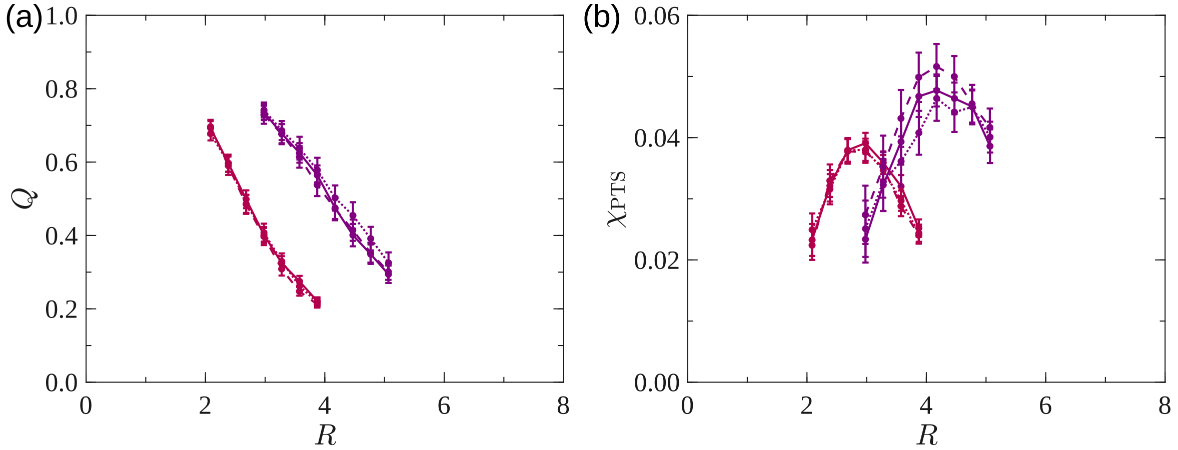

Supplementary Figure 14 PTS observables at $T = 0.101$ and 0.050 for soft disks, measured for bulk system sizes $N = 300$ (dashed), 1000 (solid), and 8000 (dotted). The color scheme is the same as in Supplementary Figure 11. (a) Radial decay of the cavity PTS correlation. (b) PTS susceptibilities with cavity radius R. The errorbars correspond to the 95% confidence intervals estimated over cavity-to-cavity variations.

R	1.5	1.8	2.1	2.4	2.7	3.0	3.3
$n_{\rm ave}$	6.28	6.35	5.96	4.98	4.86	4.95	4.39
$\lambda_{\rm dec}$	0.750	0.800	0.850	0.900	0.920	0.930	0.940
$T_{\rm dec}$	0.200	0.200	0.200	0.200	0.200	0.200	0.200
$s_{\rm eq}$	1000	1000	1000	1000	1000	1000	1000
$s_{\rm prod}$	4000	4000	4000	4000	4000	4000	4000

Supplementary Table 2 Cavity PTS measurement parameters $T = 0.200$, with 100 cavities

R	1.8	2.1	2.4	2.7	3.0	3.3	3.6
$n_{\rm ave}$	6.85	6.21	5.13	5.00	5.00	4.98	4.98
$\lambda_{\rm dec}$	0.800	0.850	0.900	0.920	0.930	0.940	0.945
$T_{\rm dec}$	0.140	0.140	0.140	0.140	0.140	0.140	0.140
$s_{\rm eq}$	1000	1000	1000	1000	1000	1000	1000
$s_{\rm prod}$	4000	4000	4000	4000	4000	4000	4000

Supplementary Table 3 Cavity PTS measurement parameters $T = 0.140$, with 100 cavities

R	2.1	2.4	2.7	3.0	3.3	3.6	3.9
n_{ave}	6.94	5.93	5.81	5.83	5.68	5.93	5.96
λ_{dec}	0.850	0.900	0.920	0.930	0.940	0.945	0.950
T_{dec}	0.125	0.125	0.125	0.125	0.125	0.125	0.125
s_{eq}	1000	1000	1000	1000	1000	1000	1000
s_{prod}	4000	4000	4000	4000	4000	4000	4000

Supplementary Table 4 Cavity PTS measurement parameters $T = 0.101$, with 100 cavities

R	2.4	2.7	3.0	3.3	3.6	3.9	4.2
n_{ave}	6.90	6.72	6.93	6.94	7.03	7.15	7.92
λ_{dec}	0.900	0.920	0.930	0.940	0.945	0.950	0.950
T_{dec}	0.125	0.125	0.125	0.125	0.125	0.125	0.125
s_{eq}	1000	1000	1000	1000	1000	1000	1000
s_{prod}	4000	4000	4000	4000	4000	4000	4000

Supplementary Table 5 Cavity PTS measurement parameters $T = 0.070$, with 100 cavities

Results for hard disks are presented in Supplementary Figure 15. Most technical details are the same as for the soft-disk case. The most notable difference concerns the parallel-tempering algorithm, which is adapted from that for $d = 3$ hard spheres [17], treating the two replicas $a = 1$ and 2 differently from the rest. Randomized configurations are here prepared by 10^6 MC sweeps with shrunk particles at $\lambda = 0.5$, and λ_{dec}s are chosen appropriately (see

R	3.0	3.3	3.6	3.9	4.2	4.5	4.8	5.1
n_{ave}	7.91	8.01	8.41	8.97	9.43	10.04	10.79	11.27
λ_{dec}	0.930	0.940	0.945	0.950	0.950	0.950	0.950	0.950
T_{dec}	0.125	0.125	0.125	0.125	0.125	0.125	0.125	0.125
s_{eq}	1000	1000	1000	1000	1000	1000	1000	1000
s_{prod}	4000	4000	4000	4000	4000	4000	4000	4000

Supplementary Table 6 Cavity PTS measurement parameters $T = 0.050$, with 100 cavities

R	3.3	3.6	3.9	4.2	4.5	4.8	5.1	5.4	5.7	6.0
n_{ave}	8.955	9.49	10.025	10.71	11.41	12.15	12.895	13.58	14.27	15.025
λ_{dec}	0.940	0.945	0.950	0.950	0.950	0.950	0.950	0.950	0.950	0.950
T_{dec}	0.125	0.125	0.125	0.125	0.125	0.125	0.125	0.125	0.125	0.125
s_{eq}	1000	1000	1000	1000	1000	1000	1000	1000	1000	1000
s_{prod}	4000	4000	4000	4000	4000	4000	4000	4000	4000	4000

Supplementary Table 7 Cavity PTS measurement parameters $T = 0.039$, with 200 cavities

R	3.9	4.2	4.5	4.8	5.1	5.4	5.7	6.0	6.3	6.6
n_{ave}	10.38	11.13	11.975	12.815	13.51	14.30	15.02	15.80	6.61	6.915
λ_{dec}	0.950	0.950	0.950	0.950	0.950	0.950	0.950	0.950	0.980	0.980
T_{dec}	0.125	0.125	0.125	0.125	0.125	0.125	0.125	0.125	0.050	0.050
s_{eq}	1000	1000	1000	1000	1000	1000	1000	1000	3000	3000
s_{prod}	4000	4000	4000	4000	4000	9000	9000	9000	27000	27000

Supplementary Table 8 Cavity PTS measurement parameters $T = 0.035$, with 200 cavities

Supplementary Tables 12-16) so that at least 96% of all the cavities pass the convergence test for all packing fractions and radii, with disorder-averaged values converging within ± 0.01. The peak location, $\xi_{\mathrm{PTS}}^{\mathrm{peak}}$, is here estimated through polynomial extrapolation of three maximal values.

R	3.9	4.2	4.5	4.8	5.1	5.4	5.7	6.0	6.3	6.6
n_{ave}	10.78	11.495	12.305	13.085	13.96	14.74	15.48	16.25	7.03	7.305
λ_{dec}	0.950	0.950	0.950	0.950	0.950	0.950	0.950	0.950	0.980	0.980
T_{dec}	0.125	0.125	0.125	0.125	0.125	0.125	0.125	0.125	0.050	0.050
s_{eq}	1000	1000	1000	1000	1000	1000	1000	1000	3000	3000
s_{prod}	4000	4000	4000	4000	4000	9000	9000	9000	27000	27000

Supplementary Table 9 Cavity PTS measurement parameters $T = 0.033$, with 200 cavities

R	3.9	4.2	4.5	4.8	5.1	5.4	5.7	6.0	6.3	6.6
n_{ave}	10.935	11.75	12.54	13.415	14.15	14.955	15.79	16.575	7.355	7.77
λ_{dec}	0.950	0.950	0.950	0.950	0.950	0.950	0.950	0.950	0.980	0.980
T_{dec}	0.125	0.125	0.125	0.125	0.125	0.125	0.125	0.125	0.050	0.050
s_{eq}	1000	1000	1000	1000	1000	1000	1000	2000	4000	4000
s_{prod}	4000	4000	4000	4000	4000	4000	9000	18000	26000	26000

Supplementary Table 10 Cavity PTS measurement parameters $T = 0.0315$, with 200 cavities

R	4.2	4.5	4.8	5.1	5.4	5.7	6.0	6.3	6.6
n_{ave}	12.243	13.147	14.027	14.893	15.717	16.643	17.41	8.2767	8.6867
λ_{dec}	0.950	0.950	0.950	0.950	0.950	0.950	0.950	0.980	0.980
T_{dec}	0.125	0.125	0.125	0.125	0.125	0.125	0.125	0.050	0.050
s_{eq}	1000	1000	1000	1000	1000	1000	2000	5000	8000
s_{prod}	4000	4000	4000	4000	9000	9000	18000	25000	32000

Supplementary Table 11 Cavity PTS measurement parameters $T = 0.028$, with 300 cavities

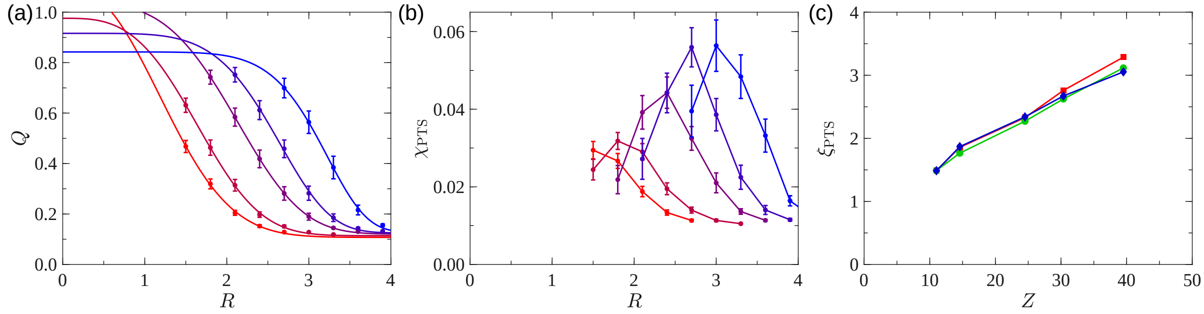

Supplementary Figure 15 : (a) Radial decay of the cavity PTS correlation at the area fraction $\varphi = 0.700, 0.740, 0.800, 0.820, 0.840$ (from red to blue) for hard disks. The errorbars correspond to the 95% confidence intervals estimated over cavity-to-cavity variations. Solid lines are fits to a compressed exponential. (b) PTS susceptibilities with cavity radius R. The errorbars are the same as in (a). Solid lines are guides for the eyes. (c) PTS lengths $\xi_{\mathrm{PTS}}^{\mathrm{fit}}$ (red-square), $\xi_{\mathrm{PTS}}^{\mathrm{th}}$ (green-circle), and $\xi_{\mathrm{PTS}}^{\mathrm{peak}}$ (blue-diamond) as a function of the reduced pressure Z for hard disks. The clear linear growth of ξ_{PTS} with Z suggests that $T_{\mathrm{K}} = 0$ with RFOT exponent $\theta = 1 = \frac{d}{2} = d - 1$ in $d = 2$ spatial dimension.

R	1.5	1.8	2.1	2.4	2.7
n_{ave}	9.14	8.88	8.08	6.57	6.00
λ_{dec}	0.750	0.800	0.850	0.900	0.920
t_{rec}	200	200	200	200	200
s_{eq}	1000	1000	1000	1000	1000
s_{prod}	4000	4000	4000	4000	4000

Supplementary Table 12 Cavity PTS measurement parameters $\varphi = 0.700$, with 100 cavities

R	1.5	1.8	2.1	2.4	2.7	3.0	3.3
n_{ave}	9.45	9.44	8.44	6.94	6.49	6.32	6.02
λ_{dec}	0.750	0.800	0.850	0.900	0.920	0.930	0.940
t_{rec}	10^4	10^4	10^4	10^4	10^4	10^4	10^4
s_{eq}	1000	1000	1000	1000	1000	1000	1000
s_{prod}	4000	4000	4000	4000	4000	4000	4000

Supplementary Table 13 Cavity PTS measurement parameters $\varphi = 0.740$, with 100 cavities

Supplementary Note 4. SCALING

The fitting result in Figure 2(b) of the main text suggests that s_{conf} and $1/\xi_{\mathrm{PTS}}$ show a linear vanishing toward zero temperature. This behaviour implies a scaling, $\xi_{\mathrm{PTS}} \propto 1/s_{\mathrm{conf}} \propto A/T + B$, where A and B are constants. Supplementary Figure 16 shows $1/s_{\mathrm{conf}}$ and ξ_{PTS}

R	1.8	2.1	2.4	2.7	3.0	3.3	3.6
n_{ave}	10.56	9.69	8.08	8.96	7.51	7.25	7.30
λ_{dec}	0.800	0.850	0.900	0.900	0.930	0.940	0.945
t_{rec}	10^4	10^4	$2 \cdot 10^4$	$2 \cdot 10^4$	$2 \cdot 10^4$	10^4	10^4
s_{eq}	1000	1000	1000	1000	1000	1000	1000
s_{prod}	4000	4000	4000	4000	4000	4000	4000

Supplementary Table 14 Cavity PTS measurement parameters $\varphi = 0.800$, with 100 cavities

R	2.1	2.4	2.7	3.0	3.3	3.6	3.9
n_{ave}	10.29	8.65	8.27	8.45	8.11	8.17	8.22
λ_{dec}	0.850	0.900	0.920	0.930	0.940	0.945	0.950
t_{rec}	10^4	10^4	$3 \cdot 10^4$	$3 \cdot 10^4$	$3 \cdot 10^4$	10^4	10^4
s_{eq}	1000	1000	1000	1000	1000	1000	1000
s_{prod}	4000	4000	4000	4000	4000	4000	4000

Supplementary Table 15 Cavity PTS measurement parameters $\varphi = 0.820$, with 100 cavities

R	2.7	3.0	3.3	3.6	3.9	4.2
n_{ave}	17.31	15.10	15.57	16.83	18.09	16.15
λ_{dec}	0.800	0.860	0.870	0.870	0.870	0.900
t_{rec}	$5 \cdot 10^4$	$5 \cdot 10^4$	$5 \cdot 10^4$	$5 \cdot 10^4$	$3 \cdot 10^4$	$2 \cdot 10^4$
s_{eq}	1000	1000	2000	2000	1000	1000
s_{prod}	4000	4000	8000	8000	4000	4000

Supplementary Table 16 Cavity PTS measurement parameters $\varphi = 0.840$, with 100 cavities

normalized at $T = 0.05$ as a function of the inverse of the temperature. The scaling relation works well in a broad range of temperature. The behavior of ξ_{PTS} can be understood as $\xi_{\text{PTS}} \sim |T - T_{\text{K}}|^{-\nu}$ with $T_{\text{K}} = 0$ and $\nu = 1$ in $d = 2$. This scaling relation is converted to $s_{\text{conf}} \propto T/(A+BT) = A^{-1}T - A^{-2}BT^2 + \mathcal{O}(T^3)$, which means that the quadratic correction shown in the main text is due to the presence of the offset B. Therefore the quadratic correction is not problematic for the determination of the critical exponents of ξ_{PTS}.

Supplementary Note 5. RELATIONSHIP WITH RECENT $d = 2$ DYNAMICAL STUDIES

Two-dimensional systems are special in condensed matter physics. Long-wavelength, Mermin-Wagner density fluctuations then destabilize long-range positional order, and thus finite-temperature crystalline solids cannot exist. While it has nonetheless long been believed that glassiness in $d = 2$ and $d = 3$ are essentially the same [22], the putative role

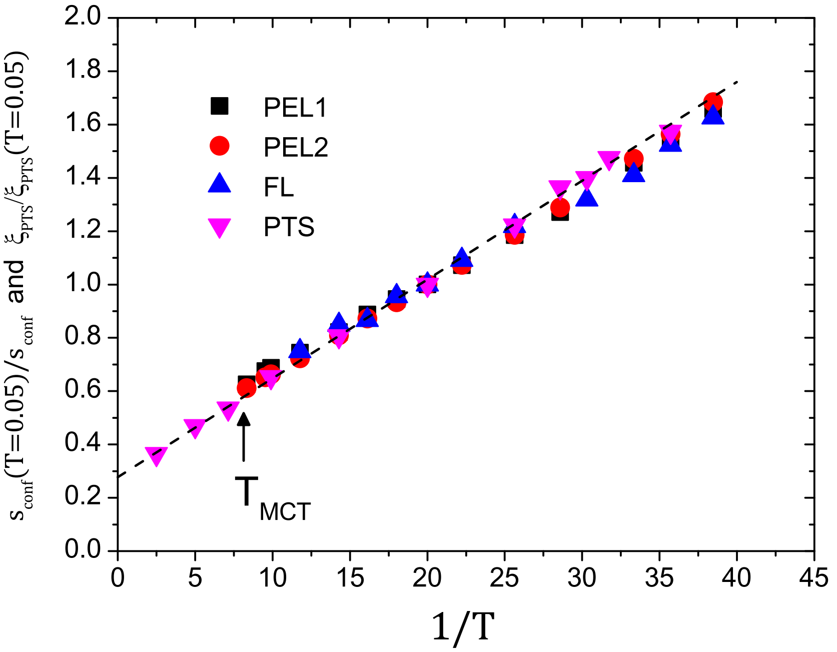

Supplementary Figure 16 : $1/s_{\rm conf}$ and $\xi_{\rm PTS}$ normalized at $T = 0.05$. The dashed line is $\xi_{\rm PTS}(T)/\xi_{\rm PTS}(T = 0.05) = A/T + B$ with $A = 0.277$ and $B = 0.037$. The vertical arrow corresponds to the mode-coupling crossover.

of Mermin-Wagner fluctuations was long neglected. Recent experimental and computational studies of glass-forming liquids have carefully considered the situation [11, 23–27]. It is now clear that, in contrast to $d = 3$, dynamics in $d = 2$ is indeed influenced by the presence of the long-wavelength density fluctuations that enhance the mean-squared displacement of particles and thus seemingly breaks the standard cage picture of glassiness. It has further been established, however, that such dynamical differences can be eliminated by studying bond-orientational relaxation or by introducing the cage-relative mean-squared displacement, which disentangles the Mermin-Wagner fluctuations from the underlying development of glassiness. Upon such disentanglement, the cage picture can be recovered in $d = 2$ as well.

Here, we disentangle Mermin-Wagner fluctuations from the measurements of the configurational entropy using approaches in the same spirit as those used in previous dynamical studies. Even after disentangling effects of these fluctuations, our determination of the configurational entropy and its comparison with $d = 3$ results suggest that the glass transition in $d = 2$ and 3 are fundamentally different. In particular, the latter occurs at a finite

temperature, whereas the former occurs at zero temperature. Our study thus identifies the lower critical dimension $d_{\mathrm{L}} = 2$ for the long-range amorphous order.

SUPPLEMENTARY REFERENCES

[1] John Russo and Hajime Tanaka, "Assessing the role of static length scales behind glassy dynamics in polydisperse hard disks," Proc. Natl. Acad. Sci. U. S. A. **112**, 6920 (2015).

[2] M. Ozawa, G. Parisi, and L. Berthier, "Configurational entropy of polydisperse supercooled liquids," J. Chem. Phys. **149**, 154501 (2018).

[3] B. Coluzzi, G. Parisi, and P. Verrocchio, "Lennard-Jones binary mixture: a thermodynamical approach to glass transition," J. Chem. Phys. **112**, 2933 (2000).

[4] M. Ozawa, W. Kob, A. Ikeda, and K. Miyazaki, "Equilibrium phase diagram of a randomly pinned glass-former," Proc. Nat. Acad. Sci., U.S.A. **112**, 6914 (2015).

[5] D. Frenkel and A. J. C. Ladd, "New Monte Carlo method to compute the free energy of arbitrary solids. Application to the fcc and hcp phases of hard spheres," J. Chem. Phys. **81**, 3188 (1984).

[6] B. Coluzzi, M. Mézard, G. Parisi, and P. Verrocchio, "Thermodynamics of binary mixture glasses," J. Chem. Phys. **111**, 9039 (1999).

[7] S. Sastry, "Evaluation of the configurational entropy of a model liquid from computer simulations," J. Phys. Condens. Matter **12**, 6515 (2000).

[8] L. Angelani and G. Foffi, "Configurational entropy of hard spheres," J. Phys. Condens. Matter **19**, 256207 (2007).

[9] Misaki Ozawa, Atsushi Ikeda, Kunimasa Miyazaki, and Walter Kob, "Ideal glass states are not purely vibrational: Insight from randomly pinned glasses," Phys. Rev. Lett. **121**, 205501 (2018).

[10] H. Shiba, P. Keim, and T. Kawasaki, "Isolating long-wavelength fluctuation from structural relaxation in two-dimensional glass: cage-relative displacement," J. Phys. Condens. Matter **30**, 094004 (2018).

[11] B. Illing, S. Fritschi, H. Kaiser, C. L. Klix, G. Maret, and P. Keim, "Mermin-Wagner fluctu-

ations in 2D amorphous solids," Proc. Natl. Acad. Sci. U. S. A. **114**, 1856 (2017).

[12] F. Sciortino, "Potential energy landscape description of supercooled liquids and glasses," J. Stat. Mech. **2005**, P05015 (2005).

[13] M. Ozawa and L. Berthier, "Does the configurational entropy of polydisperse particles exist?" J. Chem. Phys. **146**, 014502 (2017).

[14] L. Berthier, P. Charbonneau, and S. Yaida, "Efficient measurement of point-to-set correlations and overlap fluctuations in glass-forming liquids," J. Chem. Phys. **144**, 024501 (2016).

[15] P. Charbonneau, E. Dyer, J. Lee, and S. Yaida, "Linking dynamical heterogeneity to static amorphous order," J. Stat. Mech. Theory Exp. **2016**, 074004 (2016).

[16] S. Yaida, L. Berthier, P. Charbonneau, and G. Tarjus, "Point-to-set lengths, local structure, and glassiness," Phys. Rev. E **94**, 032605 (2016).

[17] L. Berthier, P. Charbonneau, D. Coslovich, A. Ninarello, M. Ozawa, and S. Yaida, "Configurational entropy measurements in extremely supercooled liquids that break the glass ceiling," Proc. Natl. Acad. Sci. U. S. A. **114**, 11356 (2017).

[18] D. Frenkel and B. Smit, *Understanding Molecular Simulation* (Academic Press, New York, ed. 2., 2001).

[19] H. Fukunishi, O. Watanabe, and S. Takada, "On the Hamiltonian replica exchange method for efficient sampling of biomolecular systems: Application to protein structure prediction," J. Chem. Phys. **116**, 9058 (2002).

[20] A. Cavagna, T. S. Grigera, and P. Verrocchio, "Dynamic relaxation of a liquid cavity under amorphous boundary conditions," J. Chem. Phys. **136**, 204502 (2012).

[21] Andrés Santos, Santos B Yuste, and Mariano Lopez de Haro, "Contact values of the radial distribution functions of additive hard-sphere mixtures in d dimensions: A new proposal," J. Chem. Phys. **117**, 5785 (2002).

[22] P. Harrowell, "Nonlinear physics: Glass transitions in plane view," Nat. Phys. **2**, 157 (2006).

[23] E. Flenner and G. Szamel, "Fundamental differences between glassy dynamics in two and three dimensions," Nat. Commun. **6** (2015).

[24] H. Shiba, T. Kawasaki, and A. Onuki, "Relationship between bond-breakage correlations and four-point correlations in heterogeneous glassy dynamics: Configuration changes and vibration modes," Phys. Rev. E **86**, 041504 (2012).

[25] H. Shiba, Y. Yamada, T. Kawasaki, and K. Kim, "Unveiling dimensionality dependence of

glassy dynamics: 2d infinite fluctuation eclipses inherent structural relaxation," Phys. Rev. Lett. **117**, 245701 (2016).

[26] S. Vivek, C. P. Kelleher, P. M. Chaikin, and E. R. Weeks, "Long-wavelength fluctuations and the glass transition in two dimensions and three dimensions," Proc. Natl. Acad. Sci. U. S. A. **114**, 1850 (2017).

[27] G. Tarjus, "Glass transitions may be similar in two and three dimensions, after all," Proc. Nat. Acad. Sci., U.S.A. , 201700193 (2017).
